# The possible role of Interleukin-6 as a regulator of insulin sensitivity in patients with neuromyelitis optica spectrum disorder

**DOI:** 10.1186/s12883-021-02198-5

**Published:** 2021-04-20

**Authors:** Zhila Maghbooli, Abdorreza Naser Moghadasi, Nasim Rezaeimanesh, Abolfazl Omidifar, Tarlan Varzandi, Mohammad Ali Sahraian

**Affiliations:** Multiple Sclerosis Research Center, Neuroscience Institute, Tehran University of Medical Sciences, Tehran, Iran

**Keywords:** Neuromyelitis optica spectrum disorder, Hyperinsulinemia, Insulin resistance, Interleukin-6, Interleukin-17, NMO-IgG, AQP4, AQP4- IgG

## Abstract

**Background:**

Neuromyelitis optica spectrum disorder (NMOSD) is associated with inflammatory mediators that may also trigger downstream signaling pathways leading to reduce insulin sensitivity.

**Methods:**

We aimed to determine the risk association of hyperinsulinemia in NMOSD patients with seropositive AQP4-IgG and the serum levels of interleukin (IL)-6 and IL-17A compared with the control group.

Serum levels of metabolic (Insulin, Fasting Blood Sugar (FBS), lipid profile) and inflammatory (IL-6 and IL-17) markers were assessed in 56 NMOSD patients and 100 controls.

**Results:**

Hyperinsulinemia was more prevalent in NMOSD patients independent of age, sex and body mass index (BMI) (48.2% vs. 26%, *p* = 0.005) compared to control group. After adjusting age, sex and BMI, there was significant association between lower insulin sensitivity (IS) and NMOSD risk (95% CI: Beta = 0.73, 0.62 to 0.86, *p* = 0.0001).

Circulating levels of IL-6 and IL-17 were higher in NMOSD patients, and only IL-6 had an effect modifier for the association between lower insulin sensitivity and NMOSD risk.

**Conclusions:**

Our data suggests that inflammatory pathogenesis of NMOSD leads to hyperinsulinemia and increases the risk of insulin resistance.

**Supplementary Information:**

The online version contains supplementary material available at 10.1186/s12883-021-02198-5.

## Background

Neuromyelitis optica spectrum disorder (NMOSD) is a rare auto-inflammatory disease of the central nervous system (CNS) [1]. The prevalence of NMOSD is approximately 0.5–4 per 100,000 population and is found in a wide range of ethnic ancestry groups around the world; with highest in Africa ethnicity and lowest in white Caucasian ethnicity [2]. In Iran, few studies reported the prevalence rate in a range of 0.86–1.9 per 100,000 population [3–5]. Through clinical, immunologic and magnetic resonance imaging (MRI) features, it can be distinguished from classic multiple sclerosis (MS) [6, 7]. The disease is characterized by “demyelination of optic nerve and/or transverse myelitis which is the result of inflammation in optic nerve and the spinal cord, respectively, leading to blindness and/or paralysis” [8].

People who suffer from NMOSD are seropositive for aquaporin 4 antibody (AQP4) - astrocytopathic disease-, myelin oligodendrocyte glycoprotein antibody (MOG) - inflammatory demyelinating disease- or seronegative for both antibodies. The mean age of onset of NMOSD in patients seropositive for AQP4 is approximately 40 years and antibody is more dominant in female than male (up to 9:1) [9].

The NMO-IgG, against aquaporin-4, is the disease-specific autoantibody that has a significant role in the pathogenesis of NMOSD as distinct from MS [10]. It specifically interacts with aquaporin-4 (AQP4) found all over the brain particularly spinal cord and optic nerves as sites of its accumulation [11]. AQP4 plays a critical role in water balance of the CNS. AQP4- IgG is produced by peripheral plasmablasts/ plasma cells and circulates in blood, crosses the blood brain barrier (BBB), and enters the central nervous system. In CNS, AQP4-IgG binds to AQP4 on astrocyte feet-ends, and then activates complement-mediated astrocyte damage, and finally leads to BBB disruption and astrocyte loss with demyelination [12]. It has been suggested that the primary breakdown of the BBB due to the present of AQP4-IgG and loss of AQP-4 in astrocyte promotes the secondary influx of plasma rich in AQP4-IgG, humoral or cellular immune components [12]. Also, sustained inflammatory cascades could raise the possibility of comorbidity of other autoimmune or inflammatory disorders [13]. Clinically, examining the pathogenesis of comorbid disorders is critical to treatment strategies for NMOSD patients.

Increasing evidence explains that circulating AQP4-IgG induces pro-inflammatory cytokines like interleukin (IL)-6, − 17 and − 2, tumor necrosis factor-α (TNF- α), interferon-γ (INF-γ), and T helper (Th)-17, and Th17-related cytokines as described in the peripheral and CNS of NMOSD patients [14–16]. It may also trigger downstream signaling pathways leading to metabolic dysregulation such as insulin pathway [14–16].

Insulin is fundamental in glucose homeostasis, and its function depends on its secretion, the target tissue sensitivity, and its clearance that are affected by pro-inflammatory cytokines [17–19]. Most on humans have examined insulin sensitivity (IS) related to obesity and diabetes but some studies have implicated lower IS or higher insulin resistance (IR) concerning neurodegenerative and autoimmune disorders such as rheumatoid arthritis [20], systemic lupus erythematosus [21], Alzheimer’s [22] and MS [23, 24]. These autoimmune disorders are linked to IR by impaired immune and inflammatory systems [25].

Based on the cytokine theory of IR [26], activation of the immune system induces cytokines, chemokines, and other inflammatory signals that lead to lower insulin sensitivity [19, 27].

In this study, we aimed to investigate the circulating insulin levels in NMOSD patients and possible effect modifier roles of two important humoral immunity factors in NMOSD pathogenesis, IL-6 and IL-17A, on insulin sensitivity in NMOSD patients.

## Methods

### Study design and population

This is a case-control study designed by the Multiple Sclerosis (MS) Research Center of Tehran University of Medical Sciences. The participants of this study were NMOSD patients who referred to MS clinic of Sina hospital as a pioneer MS referral center in Tehran, Iran. All NMOSD patients met Wingerchuck et al. [28] criteria for the diagnosis.

Totally, 158 NMOSD were registered in the MS clinic; 51% seropositive for AQP4 with the ratio female to male 7:1. Only patients with seropositive AQP4-IgG were recruited in this study.

Patients with seropositive AQP4-IgG who use steroidal drugs (prednisolone, dexamethasone, and other corticosteroids) within the last 3 months were not recruited in the present study. Medical history, family history of MS or NMOSD, the age at diagnosis of NMOSD and the medications and comorbidities of each patient were checked by the patient’s medical record, and were confirmed by neurological examination.

Participants of the healthy group were recruited by announcing in health house centers from Tehran Municipality. The control group was the healthy people who had no history of inflammatory, autoimmune, and neurological disorders, and family history of MS or NMOSD. They were referred to the MS research center of Sina hospital.

The study was conducted in accordance with the Declaration of Helsinki and Ethical Committee of the National Institute for Medical Research Development of Iran (IR.NIMAD.REC.1398.159). Written informed consent was obtained from each participant.

### Biochemical analysis

Blood samples were drawn from an antecubital vein after at least 10 h of overnight fasting for all study participants. Serums fasting blood glucose (FBS), low-density lipoprotein (LDL), high-density lipoprotein (HDL), total triglyceride (TG), and total cholesterol (TC) were assessed using enzymatic colorimetric assay (Pars-Azmun kits, Iran) by an auto-analyzer (Hitachi 902, Japan) with intra-assay precision: 0.63–1.23% and inter-assay precision of 1.09 to 1.8%.

Serum levels of IL-17A (CUSABIO, China, Cat. No.: CSB-E12819h) and IL-6 (CUSABIO, China, Cat.No.IL-6: CSB-E04638h) were determined by ELISA kits according to the manufacturer’s instructions, with intra-assay precision: CV < 8%, and inter-assay precision: CV < 10%. Serum levels of insulin were determined by ELISA kit (Monobind, Netherland) according to the manufacturer’s instructions, with intra-assay precision: CV < 6.6%, and inter-assay precision: CV < 6%.

### Definition of metabolic syndrome components

We used the modified definition of the WHO criteria [29], consisting of hyperglycemia (fasting glucose ≥110 mg/dl), waist circumference ≥ 94 cm, dyslipidemia (triglycerides ≥150 mg/dl or HDL cholesterol < 40 mg/dl), or blood Pressure (BP) ≥140/90 mmHg or taking BP medication. Also, hyperinsulinemia was defined based on the upper fourth quartile of circulating insulin in healthy group [30].

As an insulin sensitivity index, we used the QUICKIE equation [31]. IS_QUICKIE_ is derived from the product of the fasting plasma glucose (FPG) and the fasting plasma insulin (FPI). IS_QUICKIE_ *= 1/((*log (Fasting Insulin (mU/L)) + log (Fasting Glucose (mg/dl))).

Long format of the International Physical Activity Questionnaire (IPAQ) was used to assess the physical activity. Following IPAQ’s guidelines, frequency and duration of physical activity were converted to Metabolic Equivalent of Tasks (MET).

### Statistical analysis

Data analysis was performed using SPSS 20.0 (SPSS Inc., Chicago, IL). Categorical and continuous variables are expressed as number (%) and mean ± standard deviation, respectively. Normality of data was examined using the Shapiro–Wilk test. HDL, TG, insulin, SAT, ALT, IL-17A, and IL-6 did not have a normal distribution, a natural log transformation was applied to correct their normality distribution. Comparisons between study groups were performed using the Student’s t-test for continuous variables and χ2 test for categorical variables (control as a reference group). Demographics and clinical parameters were presented by descriptive statistics.

Pearson’s correlation was applied to determine the correlation between circulating metabolic markers (FBS, lipid profile, SAT, ALT, and Insulin) and inflammatory markers (IL-6, and Il-17) in NMOSD patients and controls.

A multivariable logistic regression model was used to determine the independent association of insulin resistance with NMOSD. Two-tailed *P* values < 0.05 were considered significant.

## Results

### Baseline characteristics

In the present case-control study, 56 NMOSD patients with seropositive AQP4-IgG and 100 controls without family history of MS or NMOSD were involved. Participants were recruited between December 2019 and February 2020.

The baseline and clinical characteristics of study groups are summarized in Table 1. NMOSD patients did not use corticosteroid at least 3 months before sampling. Only one patient had a family history of MS in her first relative.

**Table 1:** Clinical and biochemical characteristics of the NMOSD and control groups under study

Baseline characteristics	NMOSD ***N*** = 56	Control ***N*** = 100	***p***-value^b^
Age (years)	35.89 ± 9.39	37.40 ± 6.68	0.24
Sex (Female)	50 (89.3%)	93 (93.0%)	0.42
BMI (kg/m^2^)	26.61 ± 5.15	26.82 ± 5.45	0.81
History of alcohol consumption (whisky and/or vodka)	2 (3.57%)	1 (1%)	0.26
History of smoking	3 (5.35%)	6 (6%)	0.86
Physical activity (Ln MET-min/week)	7.36 ± 2.09	7.60 ± 1.07	0.38
Duration of the disease (years)	4.16 ± 2.91	–	–
Age of onset (years)	32.28 ± 9.11	–	–
Medications-			
Azathioprine	13 (23.2%)		
Rituximab	43 (76.8%)		
Comorbidity	8 (14.28%)	–	
EDSS score^a^	3 (3.5)		
**Biochemical and laboratory analysis**			
FBS (mg/dL)	85.47 ± 6.93	90.42 ± 9.03	**0.003**
Ln.HDL (mg/dL)	3.89 ± 0.23	3.93 ± 0.20	0.29
LDL (mg/dL)	100.71 ± 71	105.97 ± 97	0.27
Ln.TG (mg/dL)	4.71 ± 0.46	4.57 ± 0.48	0.07
Chol (mg/dL)	171.75 ± 32.62	177.77 ± 34.67	0.29
Ln.Insulin (μU/L)	2.78 ± 0.88	2.12 ± 0.48	**0.0001**
Ln. SAT (U/L)	3.30 ± 0.28	3.24 ± 0.31	0.15
Ln.ALT (U/L)	2.94 ± 0.31	2.81 ± 0.45	**0.038**
Ln.IL-17 (pg/ml)	2.00 ± 1.18	1.11 ± 0.81	**0.0001**
Ln.IL-6 (pg/ml)	3.76 ± 0.74	3.34 ± 0.62	**0.008**

^a^median (IQR)

^b^Comparisons between study groups were performed using the Student’s t-test for continuous variables and χ2 test for categorical variables

Numerical variables were expressed as the mean ± SD except for EDSS (median (IQR). Categorical variables were presented as number (percentages). Values in bold indicate statistical significance (*P* < 0.05)

*ALT* alanine aminotransferase, *SAT* serum agglutination test, *BMI* body mass index, *EDSS* expanded disability status scale, *FBS* fast blood sugar, *Chol* total cholesterol, *HDL* high-density lipoprotein cholesterol, *IL-6* interlukine-6, *IL-17* interlukine-17, *LDL* low-density lipoprotein, *Ln* natural log, *MET* metabolic equivalent of task, *TG* triglyceride

HDL, TG, insulin, SAT, ALT, IL-17A, and IL-6 did not have a normal distribution, a natural log transformation was applied to correct their normality distribution

All patients were under immunosuppression therapy (Rituximab and Azathioprine); 24 patients under second line therapy (over the last two years no change).

Among NMOSD patients, 8 persons (14.28%) had at least a history of one chronic disorder; vitiligo (1 out of 56), SLE (1 out of 56), asthma (3 out of 56), type 1 diabetes (1 out of 56), arthritis (1 out of 56) and Crohn’s disease (1 out of 56).

In our population study, there were no significant differences in age, sex, and body mass index (BMI) between the two groups; NMOSD patients and controls (*P* > 0.05).

Serum levels of insulin were higher in NMOSD patients while serum levels of FBS were lower in NMOSD patients than the control group even after adjusting with age, sex and BMI (*p* = 0.0001, *p* = 0.002; respectively).

The data analysis showed no significant differences in serum levels of TG, Cho, HDL and LDL, between the two groups; NMOSD patients and controls (*p* > 0.05) (Table 1).

### Metabolic syndrome components

The prevalence rates of metabolic syndrome components are presented in Table 2. Hyperinsulinemia was approximately 1.5 times higher in NMOSD patients than the control group (in women participants, 50% in NMOSD group and 26.9% in the healthy group (Table S1)).

**Table 2:** The prevalence of metabolic syndrome components based on modified WHO criteria in NMOSD and control groups

Metabolic syndrome component	NMOSD *N* = 56	Control *N* = 100	Un-adjusted *p*-value
Hypertension (≥140/90 mmHg)	3 (5.4%)	2 (2.0%)	0.35^a^
Dyslipidemia (triglycerides ≥150 mg/dl or HDL cholesterol < 40 mg/dl)	30 (53.6%)	52 (52.0%)	0.87
Hyperglycemia (Fasting glucose ≥110 mg/dl)	0 (0.0%)	4 (4.0%)	0.29^a^
Hyperinsulinemia (forth Quartile)	27 (48.2%)	26 (26.0%)	**0.005**
waist girth ≥94 cm	11 (19.6%)	16 (16.0%)	0.56
IS_QUICKI_	0.32 ± 0.04	0.35 ± 0.02	**0.0001**

^a^Fisher exact test

Among other metabolic syndrome components, the data analysis showed no significant differences between the two groups.

In women participants, the prevalence rates of hyperglycemia, dyslipidemia, and hypertension were similar to the control group (*p* > 0.05) (Table S1). There was no separate data analysis for the male subgroup because of its small size.

In the control group, using Pearson correlation, the data analysis showed there was positive week correlation between serum levels of insulin and FBS (*p* = 0.02, r = 0.23). However, in NMOSD group, there was not any significant correlation between serum levels of insulin and FBS (*p* = 0.1).

### Insulin sensitivity in NMOSD patients

Insulin sensitivity (Quicki index) was calculated based on fasting serum levels of insulin and FBS. There was significant lower IS in patients with NMOSD compared with the control group (0.32 ± 0.04 vs. 0.35 ± 0.02, *p* = 0.0001). In logistic regression model, after adjusting for age, sex and BMI, there was significant association between lower IS and NMOSD risk (95% CI: Beta = 0.73, 0.62 to 0.86, *p* = 0.0001).

In NMOSD patients, there were not any significant correlation between IS and duration of disease (*p* = 0.08) and EDSS score (*p* = 0.27). Also, there was not any significant difference in serum levels of insulin or insulin sensitivity in NMOSD patients with different type of medication; rituximab and azathioprine (*p* = 0.91, *p* = 0.40; respectively).

### Inflammatory markers

The data showed that serum levels of IL-17A were significantly higher in NMOSD patients than the control group (mean Ln-IL17A ± SD: 2.00 ± 1.18 pg/ml vs. 1.11 ± 0.81 pg/ml, *p* = 0.0001, respectively) even after adjusting age, sex and BMI (95% CI, beta: 2.35, 1.63 to 3.37, *p* = 0.0001).

The serum levels of IL-6 were significantly higher in NMOSD patients compared to controls (mean Ln-IL6 ± SD: 3.76 ± 0.74 pg/ml vs. 3.34 ± 0.62 pg/ml, *p* = 0.008, respectively) even after adjusting with age, sex and BMI (95% CI, beta = 2.39, 1.23 to 4.67, *p* = 0.01).

In NMOSD group, there was only significant positive correlation between IL-6 and TG (*p* = 0.025; r = 0.341). In the control group, there was only significant correlation between IL-6 and IL-17A with TG (*p* = 0.04; r = 0.322, *p* = 0.001; r = 0.338, respectively).

There were not any correlations between IL-6 and IL-17A with liver enzymes in each group (Table 3).

**Table 3:** Correlation between biochemical and inflammatory markers in NMOSD patients and control group

	NMOSD				control			
	Ln. IL-6 (pg/ml)		Ln.IL-17A(pg/ml)		Ln. IL-6 (pg/ml)		Ln.IL-17A(pg/ml)	
	r^b^	*P*-value	R	*P*-value	r^b^	*P*-value	r	*P*-value
Ln.insulin (μU/L)	**0.32**	**0.03**^a^	0.19	0.15	−0.05	0.75	−0.02	0.81
FBS (mg/dL)	0.11	0.54	0.003	0.98	0.07	0.66	0.16	0.10
Ln.TG (mg/dL)	**0.34**	**0.02**^a^	0.16	0.23	0.32	**0.04**^a^	0.34	**0.001**^a^
Ln.HDL (mg/dL)	−0.27	0.08	−0.02	0.88	−0.03	0.83	−0.06	0.56
LDL (mg/dL)	0.05	0.74	0.02	0.86	0.11	0.51	−0.008	0.93
Chol. (mg/dL)	0.003	0.98	0.03	0.84	0.16	0.34	0.10	0.32
Ln.AST (U/L)	0.12	0.44	0.19	0.18	−0.02	0.89	0.11	0.26
Ln.ALT (U/L)	−0.08	0.62	−0.03	0.81	0.06	0.73	−0.11	0.28

^a^ After adjusting with BMI, age and sex, there was constant significant correlation

^b^r: Pearson’s correlation was applied to determine the correlation between circulating markers and inflammatory markers in NMOSD patients and controls

*ALT* alanine aminotransferase, *SAT* serum agglutination test, *Chol* total cholesterol, *HDL* high-density lipoprotein cholesterol, *IL-6* interleukin −6, *IL-17A* interleukin-17A, *LDL* low-density lipoprotein, *Ln* natural log, *TG* triglyceride

There was not any significant correlation between inflammatory markers and duration of diseases in NMOSD patients (*P* > 0.12) and EDSS score (*p* > 0.34).

Regarding different type of medications, rituximab and azathioprine, there was not any significant differences in serum levels of IL-6, IL-17A and insulin in NMOSD patients (*p* = 0.81, *p* = 0.91, *p* = 0.44); respectively) (Table S2).

Among NMOSD, 60.7% (*N* = 34) were under first line therapy. There were not any significant differences in circulating levels of IL-6, IL17A and insulin in NMOSD patients under first line therapy compared ones under second line therapy (Ln-IL6: 3.91 ± 0.71 vs. 3.69 ± 0.76, *p* = 0.64, Ln-IL17A: 2.06 ± 1.31 vs. 2.09 ± 1.05, *p* = 0.92; respectively). The results were consistence even after adjusting for age and the duration of disease.

Also, there were not any significant correlation between IL-6, IL-17A with disease statues (duration of disease and EDSS) (*p* > 0.12).

### Correlation between circulating levels of inflammatory markers and insulin sensitivity

In unadjusted data analysis, there were significant correlations between insulin levels and IL-6 (*p* = 0.037, r = 0.31) but not with IL-17A (*p* = 0.15) in NMOSD patients.

In the control group, there was not any significant correlation between insulin levels and inflammatory markers, IL-6 (*p* = 0.75) and IL-17A (*p* = 0.81).

In logistic regression model, after adjusting with age, sex, BMI, and medications, there was only an independent direct association between IL-6 and NMOSD risk (95% CI: beta = 2.29, 1.06 to 4.96, *p* = 0.035). There was not any constant association between IS and NMOSD risk (*p* = 0.08).

To examine the effect modifier role of IL-17A, logistic regression model was used. After adjusting with age, sex, BMI, and taking medicines, there was independent direct association between IL-17A (95% CI: Beta = 2.15, 1.42–3.26, *p* = 0.0001) and lower IS (*p* = 95% CI: beta = 0.8, 0.68 to 0.93, *p* = 0.004) with NMOSD risk.

## Discussion

The same principle of CNS microenvironment that contributes to pathogenesis of NMOSD could promote “metabolomic perturbations” in patients. In our study, 48.2% of NMOSD patients were at risk of hyperinsulinemia compared with 26% in the control group with OR = 2.65. In line with our findings, a few studies have assessed insulin sensitivity or resistance in MS patients but not in the patients with NMOSD. Oliveira et al. revealed 40% prevalence of IR in MS patients compared with 21.1% in the control group with OR = 2.48 [23]. In another study, Ruiz-Arguelles et al. reported that insulin resistance was more prevalent in patients with MS, especially in patients with the secondary progressive type of MS [24], which had direct association with the patients’ disability levels. In newly diagnosed patients with MS, Penesova et al. observed lower insulin sensitivity in MS patients in comparison with controls [32]. Soliman et al. reported higher prevalence of insulin resistance and a three-fold prevalence of metabolic syndrome in MS patients [33]. The authors mentioned that all components of metabolic syndrome were more prevalent in women with MS.

Impairment of immune and inflammatory homeostasis is thought major drivers of the MS and NMOSD pathology. In context of NMOSD, one possible explanation for high frequency of IR in NMOSD patients is an imbalance in activated inflammatory mediators and the number and function of Th17 cells/Tregs. Although the exact cause of NMOSD remains unknown, experimental and clinical studies have showed that T cells; AQP4-specific cells, Th17 cells and Th17 cell-related cytokines, play an important role in pathogenesis of NMOSD, which were reviewed by Passos and et al. [34]. High frequency of Th17 cells and elevated levels of Th17 cell-related cytokines in NMO patients could induce CNS inflammatory cytokines in CNS lesions [34]. Inflammatory mediators that interfere with metabolic processes, can dysregulate insulin signaling and promote insulin resistance [35–37].

Regarding to insulin resistance, Tao and et al. considered the role of Th17/Treg cell balance in the development and progression of IR in Wistar rats [38]. They confirmed that Th17 cell markers such as RORγt and IL-17 and IL-6 were increased, and Treg markers such as IL-10, FoxP3 and CD4 + CD25 + Tregs were decreased. They showed the inhibition of IL-6 resulted in increased IL-10 expression and decreased IL-6 expression, reduced levels of Th17 cell markers, and an increase in Treg markers.

Interleukin-6 is an interesting cytokine with several functions in the immune system and metabolic regulation of immune responses [39]. Within the immune system, IL-6 is secreted by immunocytes and activated astrocytes [40]. It plays a key part in the adaptive immune response in NMOSD pathogenesis [41–43] by stimulating immunoglobulin (Ig) synthesis in activated B cells and Th17 cells or cytotoxic T cells development [42, 44–47]. Within the metabolic pathways, it has impairing effects on the insulin signaling which could result in IR and related diseases [39, 48] via NF-kB signaling. Increasing circulating levels of Il-6 could cross-talk with NF-kB signaling in stress oxidative condition that considered has a key role in development of some autoimmune disorders [49]. IL-6 alone or synergist with other inflammatory cytokines like TNF-α induces NF-κB activation, and thereby could promote nitric oxide synthesis that has a critical role in IR.

Iinterleukin-17 which is believed to play a key role in the inflammation that occurs in people with NMOSD leading to damage and disability. Several reports have revealed elevated CSF and blood IL-17 levels in NMO patients [40]. In inflammation sites, IL-17 produced by Th17 cells induces production of proinflammatory cytokines like TNF-α, IL-1β and IL-6 [40]. Thus, in the context of NMOSD, elevation of IL-17 levels may lead to enhanced recruitment of granulocyte to the CNS lesions, and developing disease relapse [50, 51]. In the context of metabolic regulation, IL-17 plays several roles in adipocyte differentiation, and insulin and glucose homeostasis [52]. In vivo studies propose IL-17 significant contribution in the systemic homeostasis of glucose as well [52, 53]. It is important to underline that lower IL-17 was related to a reduction in serum insulin. Reduced basal levels of insulin often represent improvement in insulin sensitivity, which may contribute to the more efficient glucose metabolism observed. Our data show that higher IL-6 and IL-17A in NMOSD patients with seropositive AQP4 while only IL-6 had a modulatory role in the course of lower insulin sensitivity. It is likely IL-17A has a modifier effect on insulin sensitivity by IL-6.

Activated IL-6 signaling may have direct effect on cell pathways like insulin in NMOSD patients. Direct targeting of IL-6 signaling may have a direct effect on plasma cell survival, autoantibody production, and restore the Th17 and Treg cells imbalance in NMOSD pathology.

However, the effect modifier role of IL-6 does not imply a causality role for IL-6 in insulin resistance in NMOSD patients. It is possible that IL-6 in combination with other inflammatory markers such as IL-1b, TNF-a, IL-18 and IL-17 links insulin resistance to NMOSD risk.

Of note, in our study population, only hyperinsulinemia was more prevalent in NMOSD patients and there were not any significant differences in other metabolic syndrome components between NMOSD patients and healthy people. As expected, hyperinsulinemia is primarily associated with impaired glucose homeostasis, but our data did not show any significant correlation between insulin levels and FBS in NMOSD patients unlike the control group. It is likely that hyperinsulinemia is associated with impaired glucose tolerance in NMOSD patients, not with fasting blood sugar, that will be examined by oral glucose tolerance test. Nevertheless, more probably it is associated with inflammatory factors identified as pathogenesis of NMOSD.

As far as we know, the present study is a first study that shows insulin resistance in NMOSD patients. However, some limitations of our study are worth noting. Firstly, our patients have used corticosteroids intermittently during the course of the disease, which might have affected insulin secretion and sensitivity. However, not all patients were on the corticosteroid as a part of treatment strategy except patients on the Rituximab who received 100 mg methylprednisolone every 6 months in combination with Rituximab. To control the effect of using the corticosteroid on insulin levels, we included patients who did not use corticosteroid 3–4 months prior to sampling.

Secondly, this was a case-control study and considered only the association between lower IS and NMOSD risk. However, based on evidence, it is likely that the pathogenesis of NMOSD might have caused higher insulin level and insulin resistance as a result.

Thirdly, 14% of our NMOSD patients had comorbidities other autoimmune disorders (vitiligo, SLE, asthma, type 1 diabetes, arthritis and Crohn’s disease), and because of the smaller sample size, it was not adjusted in the data analysis. Nevertheless, the data analysis in patients without comorbidities showed the same result (non-included).

Lastly, we evaluated only two cytokines in association with insulin resistance. In patients who develop autoimmune responses associated with insulin resistance, additional cytokines could be useful in the evaluation.

## Conclusion

The present study revealed the independent association between lower insulin sensitivity and NMOSD risk. The association was modified by higher circulating levels of IL-6 in NMOSD patients. Our data showed no association between insulin resistance and severity and duration of the disease. As immunosuppressive therapy is the main treatment in NMO patients, it seems that the treatment does not affect insulin resistance which needs additional independent treatment. There is enough evidence demonstrating the positive effect of the blockade of some cytokines like IL-6 in the improvement of insulin sensitivity in autoimmune disorders. This line of treatment can be helpful for NMOSD patients.

## Supplementary Information

**Additional file 1: Table S1.** The prevalence of metabolic syndrome components based on modified WHO criteria in NMOSD and control groups in women participants. **Table S2.** Serum levels of IL-6, IL-17A and insulin in NMOSD patients under different medications.

## Data Availability

The data that support the findings of this study are available from Multiple Sclerosis research center of Tehran University of Medical Sciences but restrictions apply to the availability of these data, which were used under license for the current study, and so are not publicly available. Data are however available from the authors upon reasonable request and with permission of research deputy of Multiple Sclerosis research center of Tehran University of Medical Sciences.

## Abbreviations

BBB: Blood brain barrier
CNS: Central nervous system
EDSS: Expanded disability status scale
TNF- α: Tumor necrosis factor-α
IL-6: Interleukin − 6
IL-17A: Interleukin − 17A
INF-γ: Interferon-γ
IS: Insulin sensitivity
IR: Insulin resistance
IPAQ: International Physical Activity Questionnaire
MS: Multiple Sclerosis
MET: Metabolic Equivalent of Tasks
NMOSD: Neuromyelitis optica spectrum disorder
AQP4-IgG: The aquaporin 4- Immunoglobulin G
Th-17: T helper − 17
