# Supplementary material for: The possible role of Interleukin-6 as a regulator of insulin sensitivity in patients with neuromyelitis optica spectrum disorder
Source: BMC Neurol. 2021 Apr 20;21:167. doi: 10.1186/s12883-021-02198-5 (PMC8056566; doi:10.1186/s12883-021-02198-5)
Supplement: Supplementary file 1 — Additional file 1: Table S1. The prevalence of metabolic syndrome components based on modified WHO criteria in NMOSD and control groups in women participants. Table S2. Serum levels of IL-6, IL-17A and insulin in NMOSD patients under different medications. [file 12883_2021_2198_MOESM1_ESM.docx]

Table S1: The prevalence of metabolic syndrome components based on modified WHO criteria in NMOSD and control groups in women participants

| **Metabolic syndrome component** | **NMOSD**  **N=50** | **Control**  **N=93** | **p-value** |
| --- | --- | --- | --- |
| Hypertension (≥140/90 mmHg) | 4 (8.0%) | 2 (2.2%) | 0.09 |
| Dyslipidemia (triglycerides ≥150 mg/dl or HDL cholesterol <40 mg/dl) | 26 (52.0%) | 48 (51.6%) | 0.96 |
| Hyperglycemia (Fasting glucose ≥110 mg/dl) | 0 (0%) | 4 (4.3%) | 0.30* |
| Hyperinsulinemia (forth Quartile) | 25 (50%) | 25 (26.9%) | **0.006** |
| waist girth ≥94 cm | 8 (16%) | 15 (16.1%) | 0.98 |
| IS_QUICKI_ | 0.32±0.04 | 0.35±0.03 | **0.0001** |

*Fisher exact test

Table S2:

Serum levels of IL-6, IL-17A and insulin in NMOSD patients under different medications

|  | **Azathioprine (N=13)** | **Rituximab (N=43)** | **p-value** |
| --- | --- | --- | --- |
| Ln.IL-17 (pg/ml) | 1.96±1.29 | 2.01±1.16 | 0.91 |
| Ln.IL-6 (pg/ml) | 3.82+0.74 | 3.75±0.76 | 0.83 |
| Ln.Insulin (µU/L) | 2.61±0.89 | 2.83±0.89 | 0.41 |
